# Genetic characteristics of chromosomally integrated carbapenemase gene (*bla*_NDM−1_) in isolates of *Proteus mirabilis*

**DOI:** 10.1186/s12866-024-03365-7

**Published:** 2024-06-19

**Authors:** Qingyu Wang, Kai Dong, Xudong Liu, Wanxiang Li, Qianyu Bian

**Affiliations:** 1 Department of Clinical Laboratory, Weifang People’s Hospital, Weifang, China; 2 Department of Emergency, Weifang People’s Hospital, Weifang, China; 3 Department of Hematology, Weifang People’s Hospital, Weifang, China

**Keywords:** *Proteus mirabilis*, Carbapenem-resistant enterobacteriaceae (CRE), *bla*_NDM−1_, Multidrug-resistant (MDR), Antibiotic resistance

## Abstract

**Objective:**

This study aims to conduct an in-depth genomic analysis of a carbapenem-resistant *Proteus mirabilis* strain to uncover the distribution and mechanisms of its resistance genes.

**Methods:**

The research primarily utilized whole-genome sequencing to analyze the genome of the *Proteus mirabilis* strain. Additionally, antibiotic susceptibility tests were conducted to evaluate the strain’s sensitivity to various antibiotics, and related case information was collected to analyze the clinical distribution characteristics of the resistant strain.

**Results:**

Study on bacterial strain WF3430 from a tetanus and pneumonia patient reveals resistance to multiple antibiotics due to extensive use. Whole-genome sequencing exposes a 4,045,480 bp chromosome carrying 29 antibiotic resistance genes. Two multidrug-resistant (MDR) gene regions, resembling Tn*6577* and Tn*6589*, were identified (MDR Region 1: 64.83 Kb, MDR Region 2: 85.64 Kbp). These regions, consist of integrative and conjugative elements (ICE) structures, highlight the intricate multidrug resistance in clinical settings.

**Conclusion:**

This study found that a CR-PMI strain exhibits a unique mechanism for acquiring antimicrobial resistance genes, such as *bla*_NDM−1_, located on the chromosome instead of plasmids. According to the results, there is increasing complexity in the mechanisms of horizontal transmission of resistance, necessitating a comprehensive understanding and implementation of targeted control measures in both hospital and community settings.

## Introduction

The global escalation in antibiotic resistance poses a formidable challenge to public health, particularly the resistance against carbapenem antibiotics in bacteria such as *Proteus mirabilis*, a prevalent member of the Enterobacteriaceae family [1–3]. This bacterium, naturally occurring in various environments and within host organisms, is capable of inciting infections across multiple human systems [4–6]. The advent and widespread application of antibiotics have led to an increased incidence of resistance in *Proteus mirabilis*, especially concerning carbapenems, thereby severely constraining therapeutic avenues and elevating the risk of treatment failures [7, 8]. Recognizing the gravity of this issue, the World Health Organization (WHO) has classified carbapenem-resistant Enterobacteriaceae (CRE) [9], particularly strains harboring resistance genes, as a top-tier threat to global health. This classification highlights the pressing need for in-depth research into the resistance mechanisms employed by these pathogens.

Carbapenems are the favored last resort drugs for treatment of severe multidrug-resistant gram-negative bacterial infections, yet their efficacy is waning due to the proliferation of resistance genes like *bla*_NDM−1_ [7, 8, 10]. These genes are predominantly found on plasmids, facilitating their horizontal transfer among various bacterial species and hastening the dissemination of resistance [11]. Conversely, chromosomal carriage of such resistance genes is uncommon [12, 13] and the dynamics of gene transfer in these contexts remain poorly understood, warranting further exploration. In light of this, our study zeroes in on a carbapenem-resistant strain of *Proteus mirabilis* (CR-PMI) that intriguingly harbors the *bla*_NDM−1_ gene within its chromosome. This phenomenon is relatively rare, and studies of its molecular characteristics benefit our understanding of the spread of carbapenem resistance.

One of our research objectives is to investigate the molecular mechanisms through which the *bla*_NDM−1_ gene integrates into the chromosomes of *Proteus mirabilis*. While we have not specifically assessed the impact of this mechanism on bacterial resistance and dissemination, our findings contributes the importance of understanding these molecular processes for the development of new antimicrobial strategies and controlling the spread of resistance.

## Materials and methods

### Clinical data collection

In this study, detailed inquiries were made into the patients’ medical history records during their hospitalization by accessing the hospital’s electronic medical record system. This process was aimed at collecting key data such as the patients’ basic information, reasons for hospitalization, treatment process, and the antimicrobial drugs used. All data collection and analysis were conducted in accordance with the hospital’s privacy protection policy and the requirements of the ethics review committee, ensuring the security and confidentiality of patient information.

### Strain collection

The CR-PMI strain was isolated in December 2020 from a tertiary teaching hospital located in northern China, which houses over 3,000 beds. The identification of this bacterial strain was conducted using Matrix-Assisted Laser Desorption/Ionization Time of Flight Mass Spectrometry (MALDI-TOF-MS) Vitek-MS, provided by Sysmex-bioMerieux in Marcy l’Etoile, France. For quality control purposes, *Escherichia coli* (ATCC 8739) was utilized as the reference strain.

### Antimicrobial susceptibility testing

To evaluate the susceptibility of key bacterial strains, the BD Phoenix™ M50 System (Becton, Dickinson and Company, New Jersey, USA) was employed to test 25 commonly used antimicrobial agents in clinical settings. The antibiotics tested include ampicillin (AMP), ciprofloxacin (CIP), meropenem (MEM), imipenem (IPM), ertapenem (ETP), piperacillin (PIP), cefuroxime (CXM), cefoperazone/sulbactam (CSL), cefotaxime (CTX), cefoxitin (FOX), levofloxacin (LVX), amikacin (AMK), amoxicillin-clavulanate (AMC), ampicillin-sulbactam (SAM), aztreonam (ATM), trimethoprim-sulfamethoxazole (SXT), piperacillin-tazobactam (TZP), gentamicin (GEN), cefepime (FEP), cefotetan (CTT), cefazolin (CZO), ceftazidime (CAZ), tobramycin (TOB), chloramphenicol (CHL), and ceftriaxone (CRO). The 2021 Clinical and Laboratory Standards Institute (CLSI) guidelines provided the criteria for determining the susceptibility of these agents [14]. *Escherichia coli* ATCC 25,922 served as the quality control strain in this evaluation, ensuring the accuracy and reliability of the testing process.

### Whole genome sequencing, de novo assembly, and annotation

Bacterial DNA from the CR-PMI strains was extracted using the Omega Bio-Tek kit(Doraville, GA, USA). Whole-genome sequencing was conducted on a NovaSeq 6000 sequencer (Illumina, CA, USA), with Unicycler v0.4.9 used for assembling and correcting the reads. TrimGalore v0.5.0 (https://github.com/FelixKrueger/TrimGalore). Quality analysis of the sequences was performed with FastQC (https://www.bioinformatics.babraham.ac.uk/projects/fastqc/). The CR-PMI strain also underwent long-read sequencing using the Nanopore PromethION platform(Oxford Nanopore Technologies, OX, UK), followed by a hybrid assembly using Unicycler v0.4.9 [15]. Sequence annotation and comparison involved predicting ORFs and pseudogenes through RAST 2.0 and the RefSeq database [16, 17], with online databases used for annotating resistance genes and other elements. Sequence comparisons were conducted using BLASTN. Figures of gene structures was drawn using the method provided in the DANMEL database [18].

## Results

### Clinical data and strain collection

In December 2020, a 57-year-old male patient was admitted to the infectious diseases department of our hospital due to tetanus and bacterial pneumonia. The patient underwent several invasive procedures, including tracheostomy, gastric tube insertion, subclavian central venous catheterization, fiberoptic bronchoscopy with lavage, and nail removal surgery due to a thumb injury. The antimicrobial treatment regimen included CAZ and MEM for two days, ETP for 14 days, and CSL for 9 days before bacterial isolation. After isolating the bacteria, the treatment was switched to TGC and LVX for 14 days, after which the patient showed improvement and was discharged. The total hospitalization period was 52 days, during which the patient was hospitalized in the ICU for a total of 25 days and later moved back to the infectious diseases department. A *Proteus mirabilis* bacterial strain (WF3430) was isolated from the patient’s sputum sample.

## Results of drug susceptibility and drug resistance genes testing

Antibiotic susceptibility testing revealed that the bacterial strain was resistant to AMC, SAM, AMP, PIP, CIP, CSL, LVX, IPM, ETP, CZO, FOX, CXM, CTX, CAZ, CRO, FEP, SXT, CHL, TOB, and GEN. It showed intermediate resistance to AMK and TZP, while being sensitive to MEM, ATM, and CTT (Table 1).

**Table 1:** Results of whole-genome sequencing and antimicrobial resistance profiles

Genetic Structure	Length (bp)	GC (%)	Total number of ORFs	Replicon type	Antimicrobial Resistance Genes	Antimicrobial Resistance Profiles
Chromosome	4,045,480	39.2%	3906		AAC(3)-IId, AAC(3)-IV, AAC(6’)-Ib-cr, aadA2, APH(4)-Ia, arr-3, catA4, catB3, CMY-59, CRP, bleMEL, dfrA1, dfrA12, dfrA32, EreA, Erm(42), floR, FosA3, mphA, NDM-1, OXA-1, QnrA1, rmtB, sul1, sul2, TEM-1, tet(G), tet(J)	R: AMP, CIP, IPM, ETP, PIP, CXM, CSL, CTX, FOX, LVX, AMC, SAM, SXT, GEN, FEP, CZO, CAZ, TOB, CHL, CRO I: AMK, TZP S: MEM, ATM, CTT
Plasmid						
	10,359	42.1%	8	ColE10_1	Not detected	

*: Resistant (R), Intermediate (I), Susceptible (S)

The genes sul1, blaTEM-1, aadA2, floR, and arr-3 are multicopy genes, with respective copy numbers of 4, 2, 2, 2, and 2

Long-read sequencing has revealed that the bacterial strain WF3430 comprises a single chromosome and one plasmid. The chromosome is 4,045,480 bp in length with a GC content of 39.2%, and it contains 3,096 open reading frames (ORFs). The plasmid is 10,359 bp long, with a GC content of 42.1%, and it has 8 ORFs, identified as belonging to the ColE10_1 replicon type with the accession number X01654. Further analysis of the assembled genome has uncovered the presence of 29 antibiotic resistance genes, including carbapenemase *bla*_NDM−1_, aminoglycosides (*AAC(3)-IId, AAC(3)-IV, AAC(6’)-Ib-cr, aadA2, APH(4)-Ia, arr-3, rmtB*), fluoroquinolones (*QnrA1*), β-lactams (*bla*_CMY−59_, *bla*_OXA−1_, *bla*_TEM−1_), chloramphenicol (*catA4, catB3, floR*), macrolides (*EreA, Erm(42), mphA*), trimethoprim (*dfrA1, dfrA12, dfrA32*), sulfonamides *(sul1, sul2*), tetracyclines (*tet(G), tet(J)*), and fosfomycin (*fosA3*). among them, the carbapenem-resistant gene *bla*_NDM−1_ was the only predicted carbapenem-resistant gene. The copy numbers of *sul1*, *bla*_TEM−1_, *aadA2, floR*, and *arr-3* genes were 4, 2, 2, 2, and 2 respectively. Upon comparing the antimicrobial susceptibility profiles with the genotypic data, it was observed that the majority of antimicrobial phenotypes matched the genotypes. However, the susceptibility profile revealed resistance to carbapenems, including ETP and IPM, while MEM showed sensitivity. Notably, all these resistance genes are located on the chromosome. No antibiotic resistance genes were found on the plasmid.

### Structures of chromosomally integrated MDR regions

In our study, we identified and analyzed two significant multidrug resistance (MDR) gene regions, named MDR region 1 and MDR region 2, which are located on the bacterial chromosome. These regions exhibit unique structural features and a composition of resistance genes at specific chromosomal locations.

**Fig. 1:**
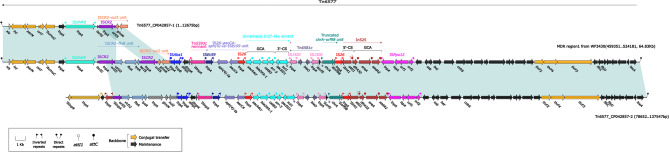
Organization of the MDR region 1 from WF3430. Genes are denoted by arrows. Genes, mobile genetic elements and other features are coloured based on their functional classification. Shading denotes regions of homology (light blue: ≥99% nucleotide identity). The accession number of Tn*6577* used as reference is CP042857-2

MDR region 1 spans from 459,351 to 524,181 on the chromosome, covering 64.83Kb. This region matches specific parts of the Tn6577 transposon, specifically from 1 to 12,675 bp and from 78,652 to 137,547 bp. Within this MDR region, we discovered an integron structure composed of two parts: the 5’-conserved segment (5’-CS) and the 3’-conserved segment (3’-CS), both located downstream of the IS*26* sequence. Additionally, the region contains multiple antibiotic resistance genes, such as *APH(4)-Ia*, *floR*, *sul2*, *bla*_OXA−1_, *catB3*, *arr-3*, *sul1*, *dfrA32*, *EreA*, and *aadA2*, along with several insertion sequences (IS). These resistance genes and IS sequences from multiple identified resistance units as depicted in Fig. 1.

**Fig. 2:**
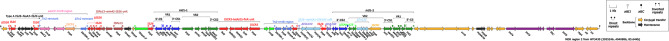
Organization of the MDR region 2 from WF3430. Genes, mobile genetic elements and other features are coloured based on their functional classification

The MDR region 2, spanning the chromosome from positions 3,955,246 to 4,040,886, encompasses an 85.64 Kbp length. This region shares partial structural similarity with Tn*6582* (9321.13445 bp) and segments of p112298-KPC (23369.26698 bp), while the majority aligns more closely with the transposon Tn*6589*, exhibiting extensive structural homology. Notably, this area incorporates two composite integrons(*intI*1-1and *intI*1-2), an array of insertion sequences (IS), and a resistance module composed of multiple antimicrobial resistance genes, as depicted in Fig. 2.

The composite integron *intI*1-1 located on the downstream of an IS*26* sequence, displaying an atypical structure where its 3’-CS2 is solely constituted by truncated versions of *sul1* and *qacED1*, absent of the *orf5* and *orf6* coding frames. This integron intI1-1 harbors variable regions carrying resistance genes such as *dfrA1*, *gcuC*, *qnrA1*, and *ampR*. Conversely, the right-side composite integron intI1-2 exhibits a structure mirroring that of the left, positioned upstream of IS*26* and a truncated IS*6100* sequence, according to the direction of translation. This integron was noteworthy for carrying the *bla*_NDM−1_ gene, pivotal for the bacterium’s resistance to carbapenem antibiotics, located within its second variable region (VR2). This variable region also integrates multiple mobile genetic elements including IS*CR1*, ΔTn*125*, and ΔISA*ba125*, thereby also encompassing genes such as *arr-3*, Δ*catB3*, and *ble*_MBL_. Additionally, the VR1 area of this integron carries resistance genes including *aadA2*, *gcuF*, and *dfrA12*, further contributing to its complex resistance profile.

MDR region 1 and MDR region 2 containing 10 and 13 IS sequences, respectively. Which typically located upstream or downstream of resistance genes, forming various known resistance units. These resistance units, along with the mentioned integron structures, define the characteristics of the two MDR regions. The annotation of the overall genetic structure for MDR region 1 and MDR region 2 indicates that both sequences include attL (attachment site at the left end of the ICE), int (integrase), xis (excisionase), oriT (origin of conjugative replication), a F (TivF)-type T4SS machinery (mating pair formation), and attR (attachment site at the right end of the ICE). Not only that, the 3’ and 5’ termini of MDR region 1was the same as the 3’and 5’ termini of Tn6577, respectively. demonstrating that both segments belong to an integrative conjugative element (ICE) structure.

## Discussion

In both hospital and community settings, *Proteus mirabilis* has become a significant causative agent of various infections [2, 19–21]. The escalating challenge posed by this bacterium is amplified by its acquired resistance to multiple antibiotics, notably including carbapenems. The surge in resistance to carbapenem antibiotics, a last resort drugs utilized for treating infections caused by multidrug-resistant gram negative bacteria, adds complexity to the already intricate landscape of clinical management [22–25]. Addressing this critical concern, the focus of this study is a meticulous examination of a clinical strain identified as CR-PMI. By undertaking an in-depth exploration of the molecular mechanisms governing its antibiotic resistance. The aim was to furnish essential data that can guide strategies for implementing effective measures for clinical infection control.

The subject of this study is an elderly male patient admitted for tetanus and bacterial pneumonia, undergoing multiple invasive treatments during the course of his medical care. This patient, being at a high risk for nosocomial infections, shares similarities with previous cases involving infections caused by *Proteus mirabilis* [2, 5, 6, 26]. This suggests a pronounced infectivity and pathogenicity of *Proteus mirabilis*, especially in immunocompromised individuals. It is worth noting that the CR-PMI strain was promptly isolated when patients was transferred from the intensive care unit to the infectious disease department, indicating the potential acquisition of CR-PMI through nosocomial transmission. This raises concerns about a potential localized outbreak of the bacterium within our healthcare facility. Emphasizing the need for comprehensive epidemiological investigations, it is crucial to conduct a thorough examination to ascertain the presence and extent of CR-PMI within our hospital setting.

In this study, the strains detected drug resistance genes containing most commonly used antimicrobial drugs, and the resistance phenotype showed highly resistance, which is consistent with the results of recent studies [27–29], which indicates that the resistance has been increasing in recent years. Moreover, a comparison of the bacterium’s resistance phenotype and genotype reveals a correlation between most drug resistances and the carried resistance genes. However, the sensitivity of this strain to meropenem (MEM) contradicts the typical phenotype of Enterobacteriaceae harboring *bla*_NDM−1_, as previous studies have indicated that *bla*_NDM−1_-positive strains are generally resistant to all carbapenems [27, 30–32]. The phenomenon of MEM sensitivity in *bla*_NDM_-positive strains [33] warrants additional experimental investigations to uncover the underlying reasons for this phenomenon.

Through whole-genome sequencing, it was discovered that all resistance genes of this bacterium, including *bla*_NDM−1_, are located on the chromosome, with the sole predicted plasmid carrying no antimicrobial resistance genes. This deviates from the conventional understanding that Carbapenemase resistance genes in Enterobacteriaceae are primarily plasmid-borne [33–35]. This peculiar characteristic might be attributed to distinct mechanism of acquire antimicrobial resistance genes in *Proteus mirabilis*, necessitating further research to enhance our understanding of its role in antibiotic resistance development.

Analysis of the sequencing data revealed that the chromosome of this bacterium carries two regions associated with resistance genes (MDR region 1 and MDR region 2). Within these regions, three copies of Class I integrons were identified, all containing numerous IS sequences closely related to the resistance genes discovered in this study. These IS sequences constitute various known resistance units, crucial for the bacterium’s resistance to multiple clinical antimicrobial drugs. In particular, IS 26, located upstream or downstream of the three integrons and some resistance genes in this study. It indicates that it should play a very important role in the horizontal transmission of drug resistance genes and integrons. Importantly, the three integron variable regions identified in this study carry multiple resistance genes, contributing to the bacterium’s resistance profile. Of significance, the *bla*_NDM−1_ gene is also found within the variable region of composite integron(intI1-2). However, its location within the second variable region (VR2) of the integron may influence its expression levels due to the distance from the promoter, as suggested by previous research on the relationship between resistance gene expression and integron promoter structure [36, 37]. Furthermore, it has been traditionally believed that integrons primarily integrate carbapenem resistance genes such as *bla*_GIM_, *bla*_VIM_, and *bla*_IMP_ into plasmids, with *bla*_NDM_ being less commonly associated with integrons, and integration into the chromosome even rarer [37–39]. This observation underscores the significant contribution of integrons to resistance development in *Proteus mirabilis* [37–39], highlighting the need for further investigation into integron structure and function.

Upon detailed annotation of the two MDR regions, we observed that both regions belong to ICE structures. Previous studies have indicated that ICEs constitute a type of mobile genetic elements (MGEs) primarily residing in the bacterial chromosome, with the ability to carry resistance genes [40]. Furthermore, ICEs can be transferred between cells through conjugation, a self-encoded function. Therefore, we propose that the composite integron containing *bla*_NDM−1_ gene may have been integrated into the ICE (MDR region 2) transposon through IS sequences, subsequently transposing into the chromosome. This mode of transfer was previously observed in *Proteus mirabilis* isolated from livestock and food sources, where the chromosome-encoded *bla*_NDM−1_ gene was reported [2, 27, 29]. However, to our knowledge, this phenomenon is not common in CR-PMI strain isolated from human specimens. Additionally, similar occurrences have been documented in recent years in other strains, as seen in carbapenem-resistant *Klebsiella pneumoniae, Escherichia coli, Acinetobacter baumannii a*nd other bacteria, where chromosome-encoded carbapenem resistance genes such as *bla*_KPC−2,_*bla*_VIM−4_ and *bla*_OXA−48_-like have been identified [12, 13, 31, 41, 42].

The emergence of such strains highlights the increasing complexity of resistance mechanisms, emphasizing the need to not only focus on resistance genes themselves and their presence on plasmids but also investigate the mechanisms by which chromosome-encoded resistance genes are formed. This is particularly crucial in the case of *Proteus mirabilis*, where multiple genetic elements collaboratively contribute to the spread of resistance mechanisms.

## Conclusion

This study found that a CR-PMI strain exhibits a unique mechanism for acquiring antimicrobial resistance genes, such as *bla*_NDM−1_, located on the chromosome instead of plasmids. Multidrug-resistant regions on the chromosome serve as mobile genetic elements carrying resistance genes belonging to ICE structures. The presence of Class I integrons and IS sequences within these regions is significant for the transfer of resistance genes. According to the results, there is increasing complexity in the mechanisms of horizontal transmission of resistance, necessitating a comprehensive understanding and implementation of targeted control measures in both hospital and community settings.

## Data Availability

The WGS sequence data of Proteus mirabilis isolate has been deposited in the NCBI database and are available under BioProject: PRJNA885151 (For short reads: SAMN31078299, accession number: JAOSFI000000000, and for long reads: SAMN39972476, accession number: CP145479.1).
